# Correlation of Hemoglobin Level With New Inflammatory Markers in the Emergency Department: A Retrospective Study Exploring Neutrophil-to-Lymphocyte, Monocyte-to-Lymphocyte, Platelet-to-Lymphocyte, and Mean Platelet Volume-to-Platelet Count Ratios

**DOI:** 10.7759/cureus.55401

**Published:** 2024-03-02

**Authors:** Majd Hassan, Charbel Abdayem, Sarine El Daouk, Bassam F Matar

**Affiliations:** 1 Department of Medicine, Faculty of Medical Sciences, Lebanese University, Beirut, LBN; 2 Department of Medicine, Faculty of Public Health 1, Lebanese University, Beirut, LBN; 3 Department of Hematology and Oncology, Lebanese University, Al-Zahraa Hospital University Medical Center, Beirut, LBN

**Keywords:** receiver operating characteristic (roc) analysis, hospital admission, emergency medical service, mean platelet volume/platelet ratio (mpv/plt ratio), platelet to lymphocyte ratio (plr), monocyte to lymphocyte ratio, neutrophil to lymphocyte ratio (nlr), correlation analysis, immune-inflammation indexes, hemoglobin: hb

## Abstract

Background

Anemia of chronic disease is known to be associated with inflammation. However, the relationship between hemoglobin (Hb) levels and potential inflammatory markers such as neutrophil-to-lymphocyte ratio (NLR), monocyte-to-lymphocyte ratio (MLR), platelet-to-lymphocyte ratio (PLR), and mean platelet volume-to-platelet count ratio (MPV/PC) has not been extensively studied. The primary objective of this retrospective analytical study conducted at Al Zahraa Hospital University Medical Center (ZHUMC), Beirut, was to investigate the correlation between Hb levels and potential inflammatory markers (NLR, MLR, PLR, MPV/PC) in patients visiting the emergency department (ED), across different genders and age groups. The secondary objectives were to compare Hb levels and inflammatory markers values between the referred medical ward group (the hospitalized patients who were admitted to the medical ward), and the non-referred to medical ward group (the patients who were discharged home from the ED), and to evaluate the predictability of inflammatory markers and Hb levels for referral to the medical ward, including the determination of optimal cutoff values for hospital admission to the medical ward.

Methods

We analyzed the blood parameters of 379 adult patients who presented to the ED with various medical complaints between September 1, 2022, and November 30, 2022 (three months). These patients were included in the study after we checked their eligibility regarding the verification of all our inclusion and exclusion criteria.

Results

Our findings revealed a significant negative correlation between Hb levels and PLR (r = -0.24) in both genders and across different age groups. The group referred to the medical ward exhibited lower Hb levels and higher NLR, MLR, and PLR values (P < 0.001). NLR/Hb ratio emerged as a predictive factor for admission in genitourinary (R² = 0.158; OR = 5.62) and respiratory groups (R² = 0.206; OR = 5.89), with specific cutoff values of 0.533 (Sensitivity = 57.1% & Specificity = 84.2%) and 0.276 (Sensitivity = 85% & Specificity = 51.1%), respectively.

Conclusions

Our study demonstrates that hemoglobin level negatively correlates with PLR. NLR, MLR, and PLR stand as important inflammatory markers. Moreover, we present the first study in the literature to show that NLR/Hb ratio can serve as a predictor for referral to the medical ward, particularly in the genitourinary and respiratory patient groups, underscoring its value in risk assessment as a prognostic marker reflecting the need for admission when the case is more serious.

## Introduction

Anemia of chronic disease (ACD), also known as anemia of inflammation, is a hypo-proliferative anemia that occurs in response to systemic disease or inflammation. It is the most common form of anemia observed in hospitalized or chronically ill patients, ranking second globally after iron deficiency anemia (IDA). ACD can manifest in various diseases characterized by an exaggerated immune response, including cancer, infections, autoimmune diseases, chronic kidney disease, heart failure, chronic lung disease, and obesity [1,2]. The pathogenesis of ACD involves the dysregulation of iron homeostasis and erythropoiesis, mediated by cytokines and acute phase proteins [3].

Given the interdependence of immunity and coagulation, as well as the association between systemic inflammation and altered peripheral blood leukocytes, novel parameters derived from routine complete blood count with differential (CBCD) have been proposed as potential inflammatory biomarkers [4,5]. These include the neutrophil-to-lymphocyte ratio (NLR), monocyte-to-lymphocyte ratio (MLR), platelet-to-lymphocyte ratio (PLR), and mean platelet volume-to-platelet count ratio (MPV/PC ratio). Numerous studies have demonstrated the discriminative ability of these emerging inflammatory markers in predicting outcomes for various solid neoplasms, such as cervical carcinoma, and gastrointestinal, kidney, and lung cancer [5,6].

While studies investigating the correlation between hemoglobin levels (Hb) and C-reactive protein levels (CRP) in the emergency department (ED) have been conducted [7], there is a notable lack of research examining the relationship between Hb and the potential inflammatory markers: NLR, MLR, PLR, and MPV/PC ratio. Additionally, the predictive ability of the CRP/Hb ratio for hospital admission has been explored, but there is a lack of studies investigating the predictive value of these potential inflammatory markers/Hb ratios and their utility in predicting hospitalization from the ED [7].

Therefore, our primary objective is to investigate the correlation between hemoglobin levels and our proposed novel inflammatory markers in patients admitted to the ED, stratified by gender and age. The secondary objectives of our study are to compare the levels of these inflammatory markers between patients referred and not referred to the medical ward, and to assess the ability of these markers, as well as the potential inflammatory makers over Hb ratios, to predict referral to medical ward within the different final medical diagnosis groups.

## Materials and methods

Participants

We conducted a retrospective analytical monocentric study by reviewing the medical records of patients who visited the ED at Al-Zahraa Hospital University Medical Center (ZHUMC) in Beirut, Lebanon. The study period spanned three months, from September 1, 2022, to November 30, 2022. After removing outliers using Statistical Package for the Social Sciences (SPSS) software, the final sample size (N) was determined to be 379. We collected the necessary information from the medical records and recorded it on a prepared Excel sheet. The variables of interest included parameters from the first complete blood count with differential (CBCD) test, such as hemoglobin levels, platelet count, mean platelet volume, absolute neutrophil count, absolute lymphocyte count, and absolute monocyte count. Additional information, such as age, gender, referral to medical ward status, and final medical diagnosis, was also recorded.

The selected patients were adults aged 18 years and older, comprising both males and females. Pregnant females and patients, both males and females, with final diagnoses related to trauma or surgery, those who left the hospital against medical advice, refused diagnostic tools for management, had incomplete CBCD tests, missing data in their files, or visited the ED multiple times during the study period were excluded.

It is worth mentioning in this section that the decision for patient admission to the hospital and referral to the medical ward is usually made by the attending physicians in the emergency department. This decision-making process takes into account various factors, including the severity of the underlying medical condition, the overall clinical status of the patient, and the adherence to established clinical guidelines.

Data collection

The required data were collected from the patient's medical records stored in the archive of ZHUMC and transferred to a prepared Excel ((Microsoft Corporation, Redmond, WA, USA) sheet. The sheet included patient IDs, medical record numbers, and the aforementioned parameters from the study design and population section. Ratios such as NLR, MLR, PLR, MPV/PC ratio, NLR/Hb ratio, MLR/Hb ratio, PLR/Hb ratio, and MPV/PC/Hb ratio were calculated using Excel. Blood samples collected from the patients were analyzed for CBCD using the Sysmex XN-1000 (Sysemex Corp., Kobe, Japan) machine in the hospital's laboratory.

Statistical analysis

The data entered into the Excel Sheet were analyzed using SPSS software, version 25, (IBM Corp., Armonk, NY). Statistical significance was determined at P < 0.05. The correlation of Hb levels with NLR, MLR, PLR, and MPV/PC ratios in the total sample, as well as in different age and gender groups, was assessed using Pearson's correlation test. Hb levels and the ratios were compared between the groups referred and not referred to the medical ward using Student's t-test.

The statistical analysis was initially conducted on the entire sample (N = 379) to determine the general correlation between Hb levels and inflammatory markers. Subsequently, a comparison was made between the referred group (n = 63) and the non-referred group (n = 316) regarding their Hb levels and inflammatory marker values. Finally, the predictive ability of Hb levels and different inflammatory markers was investigated for admission to the medical ward. This analysis was performed on the entire sample and separately for the three most prevalent final medical diagnosis groups, namely the digestive group (n = 112), genitourinary group (n = 71), and respiratory group (n = 67).

The predictive ability, sensitivity, specificity, and area under the curve (AUC) of all ratios were determined using receiver operating characteristics (ROC) analysis. The ratio with the highest Nagelkerke R-square value was selected, and Youden's index was calculated using the formula: Youden's index = specificity + sensitivity - 1. The cutoff value for referral to the medical ward was determined as the value with the best balance of sensitivity and specificity. Logistic regression was employed to calculate the p-value, exp (B), and 95% confidence interval (CI) for exp (B) for the best predictive ratio regarding referral to the medical ward.

## Results

Participants and sample characteristics

The study sample consisted of 379 patients, with 56.5% being female. Out of these patients, 16.6% were referred to the medical ward. The top three reasons for ED admission were related to digestive, genitourinary, and respiratory diseases. Furthermore, 76% of the patients in the sample were below the age of 65. These demographic and clinical characteristics provide important insights into the composition of the studied population as shown in Table 1.

**Table 1:** Descriptive frequencies of the sample characteristics Demographics and characteristics of the 379 patients

Characteristics	N = 379 (%)
Gender, n (%)	
Male	165 (43.5)
Female	214 (56.5)
Age group, n (%)	
< 65 years	288 (76)
≥ 65 years	91 (24)
Final medical diagnosis group, n (%)	
Digestive	112 (29.6)
Genitourinary	71 (18.7)
Respiratory	67 (17.7)
Cardiovascular	40 (10.6)
Psychological	34 (9)
Neurological	16 (4.2)
Musculoskeletal	9 (2.4)
Oncological	5 (1.3)
Hematological	5 (1.3)
Skin	5 (1.3)
Endocrine and metabolic	4 (1.1)
Head and neck	4 (1.1)
Immunological	4 (1.1)
Poisoning	3 (0.8)
Referral to medical ward group, n (%)	
Yes	63 (16.6)
No	316 (83.4)

Correlation between hemoglobin level and MPV/PC, NLR, MLR and PLR ratios

**Figure 1:**
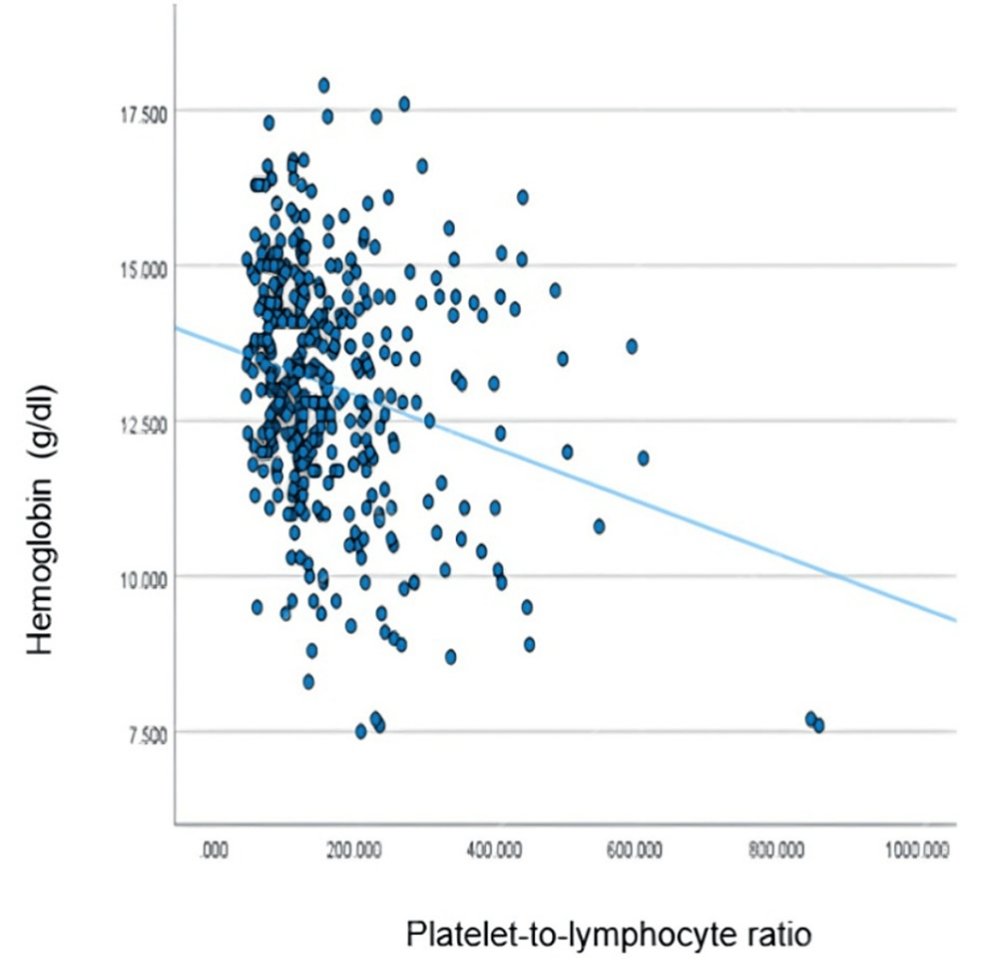
Correlation between hemoglobin and platelet-to-lymphocyte ratio in the entire sample r^2^ linear = 0.059

We observed a significant negative correlation between Hb and PLR (r = -0.24, r² = 0.059, P < 0.05) in the entire sample (Figure 1). In males, there was a significant negative correlation between Hb and MLR (r = -0.16, P < 0.05). Both gender groups showed a significant negative correlation between Hb and PLR, with a slightly stronger negative correlation observed in females (r = -0.21 and r = -0.28, respectively, P < 0.05). Additionally, there was a significant negative correlation between Hb and PLR in individuals aged < 65 years old and in those aged ≥ 65 years old (r = -0.19 and r = -0.24, respectively, P < 0.05), with the correlation being stronger in individuals aged 65 and above (Table 2).

**Table 2:** Correlation of hemoglobin levels with related ratios distributed by gender and age in the entire sample * P value < 0.05 MPV/PC ratio: mean platelet volume-to-platelet count ratio NLR: neutrophil-to-lymphocyte ratio MLR: monocyte-to-lymphocyte ratio PLR: platelet-to-lymphocyte ratio

Ratios	Hb (g/dl)				
	Total sample	Male	Female	Age < 65 years	Age ≥ 65 years
MPV/PC ratio (µm³/10³/µL)	0.01	-0.07	-0.06	0.06	-0.04
NLR	-0.04	-0.13	0.05	0.01	0.01
MLR	-0.04	-0.16*	-0.06	0.04	-0.07
PLR	-0.24*	-0.21*	-0.28*	-0.19*	-0.24*

Comparison between Hb level and the inflammatory markers between the referred and non-referred to medical ward groups

There was a significant difference in hemoglobin (Hb) levels between the referred and non-referred to medical ward groups in the entire sample (11.93 ± 2.17 vs. 13.26 ± 1.78, P < 0.001). Specifically, the referred group had lower Hb levels compared to the non-referred group.

On the other hand, neutrophil-to-lymphocyte ratio (NLR), monocyte-to-lymphocyte ratio (MLR), and platelet-to-lymphocyte ratio (PLR) were significantly higher in the referred group compared to the non-referred group (NLR: 7.53 ± 6.72 vs. 4.76 ± 4.48, MLR: 0.42 ± 0.3 vs. 0.31 ± 0.22, PLR: 216.72 ± 161.1 vs. 161.27 ± 93.87, respectively, P < 0.001).

It's worth noting that there was no significant difference in the mean platelet volume per platelet count (MPV/PC) value between the referred and non-referred to medical ward groups (0.05 ± 0.02 vs. 0.04 ± 0.01, P = 0.304) (Table 3).

**Table 3:** Comparison of Hb levels and inflammatory markers of referred vs. non-referred to medical ward groups *P value < 0.05, ** Student's t-test Hb: Hemoglobin MPV/PC ratio: mean platelet volume-to-platelet count ratio NLR: neutrophil-to-lymphocyte ratio MLR: monocyte-to-lymphocyte ratio PLR: platelet-to-lymphocyte ratio

Blood tests	Referral to medical ward (μ±SD)		P value **
	Yes (N=63)	No (N=316)	
Hb (g/dl)	11.93 ± 2.17	13.26 ±1.78	<0.001*
MPV/PC ratio (µm³/10³/µL)	0.05 ± 0.02	0.04 ±0.01	0.304
NLR	7.53 ± 6.72	4.76 ± 4.48	<0.001*
MLR	0.42 ± 0.30	0.31 ± 0.22	<0.001*
PLR	216.72 ± 161.1	161.27 ± 93.87	<0.001*

Predictive ability of NLR/Hb ratio regarding admission to medical ward in the entire sample and in the most prevalent groups

Of all the studied potential inflammatory markers NLR, MLR, PLR, MPV/PC ratio, and their ratios with hemoglobin notably NLR/Hb ratio, MLR/Hb, PLR/Hb, and MPV/PC/Hb ratios, NLR/Hb ratio was the most predictive for referral to medical ward in the entire sample, having the highest Nagelkerke R Square value equal to 0.098 meaning this model can explain 9.8% of the variation of NLR/Hb ratio in the entire sample. Thus, we used the NLR/Hb ratio to check its predictive ability regarding admission to the medical ward also in the three most prevalent groups.

Logistic regression of NLR/Hb ratio in the entire sample and in the three most prevalent groups

In our logistic regression analysis, we examined the predictive ability of the NLR/Hb ratio for admission to the medical ward. We found that patients presenting to the ED with an NLR/Hb ratio greater than 0.289 were 4.01 times more likely to be admitted to the medical ward (95% CI {2.25, 7.13}, P < 0.001).

This finding was consistent across the entire sample and in the three most prevalent diagnostic groups. In the digestive group, the contribution of the NLR/Hb ratio to the model was not statistically significant (P = 0.089). However, in the genitourinary group, the NLR/Hb ratio showed a significant contribution to the model (P = 0.01), as well as in the respiratory group (P = 0.004). These results led us to select the genitourinary and respiratory groups for further analysis, considering their significant associations with the NLR/Hb ratio.

Additionally, we found patients with a final diagnosis related to the genitourinary system and an NLR/Hb ratio greater than 0.533 were 5.62 times more likely to be admitted to the medical ward (95% CI {1.52, 20.76}, P = 0.01). Similarly, patients with a final diagnosis related to the respiratory system and an NLR/Hb ratio greater than 0.276 were 5.89 times more likely to be admitted to the medical ward (95% CI {1.74, 19.85}, P = 0.004) (Table 4).

**Table 4:** Contribution of NLR/Hb ratio to the model for admission to the medical ward, analysis in the entire sample, digestive group, genitourinary group, and respiratory group NLR/Hb ratio: neutrophil-to-lymphocyte over hemoglobin ratio N/A: not applicable

Performed logistic regressions	Ratios	Significance	Exp (B)	95% CI for Exp (B) Lower	95% CI for Exp (B) Upper
Entire sample	NLR/Hb ratio (dl/g)	< 0.001	4.01	2.25	7.13
	Constant	< 0.001	0.10	N/A	N/A
Digestive group	NLR/Hb ratio (dl/g)	0.089	2.63	0.86	8.03
	Constant	< 0.001	0.84	N/A	N/A
Genitourinary group	NLR/Hb ratio (dl/g)	0.01	5.62	1.52	20.76
	Constant	< 0.001	0.11	N/A	N/A
Respiratory group	NLR/Hb ratio (dl/g)	0.004	5.89	1.74	19.85
	Constant	< 0.001	0.15	N/A	N/A

ROC analysis, Youden’s indexes, and cutoff calculation for NLR/Hb ratio in the entire sample and in the genitourinary and respiratory groups

Using ROC analysis, we determined the cutoff value of the NLR/Hb ratio for referral to the medical ward. The cutoff value was selected based on achieving the best balance between sensitivity and specificity. In the entire sample, the cutoff value was found to be 0.289, with a sensitivity of 68.3% and a specificity of 59.8% (AUC = 0.661).

In the genitourinary group, the cutoff value for the NLR/Hb ratio was determined to be 0.533, with a sensitivity of 57.1% and a specificity of 84.2% (AUC = 0.724). Similarly, in the respiratory group, the cutoff value was found to be 0.276, with a sensitivity of 85% and a specificity of 51.1% (AUC = 0.719).

**Figure 2:**
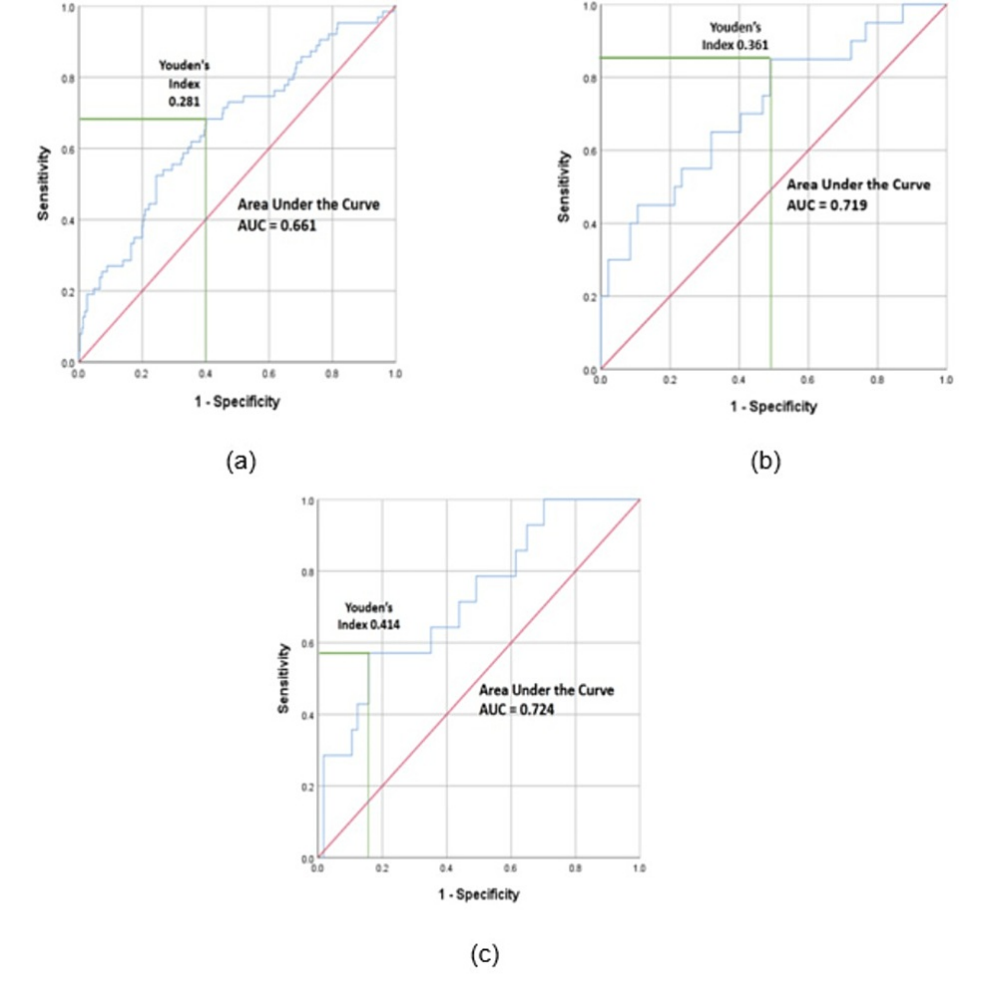
ROC curves, AUC, and Youden's indexes of NLR/Hb ratio in the entire sample, respiratory and genitourinary groups (a): Entire sample (b): Respiratory group (c): Genitourinary group ROC: receiver operating characteristic AUC: area under the curve NLR/Hb ratio: neutrophil-to-lymphocyte over hemoglobin ratio

Additionally, Youden's index was 0.281 in the entire sample, while in the respiratory group, it was 0.361, and in the genitourinary group it was 0.441. These indices provide an overall measure of the diagnostic accuracy of the NLR/Hb ratio in each group (Figure 2).

## Discussion

To the best of our knowledge, our study is the first to demonstrate a negative correlation between Hb and PLR as well as the introduction of NLR/Hb ratio as a predictive marker for referral to the medical ward. The ratios NLR, MLR, PLR, and MPV/PC ratio can be easily obtained from a simple CBCD, making them affordable and readily available for clinical use.

The NLR is a simple ratio calculated by dividing the absolute neutrophil count to the absolute lymphocyte count. An increase in neutrophil count and a decrease in lymphocyte count both reflect the body’s natural physiological response to systemic inflammation. Inflammatory cytokines promote the massive recruitment of neutrophils (through activating and chemotaxis) and cause a reduction in circulating lymphocytes (through depletion and consumption) [8].

MLR is calculated by dividing the absolute monocyte count by the absolute lymphocyte count. It is a novel inflammatory marker with prognostic ability for many cancers. Inflammatory responses stimulate the release of monocytes from the bone marrow into circulation, and activated monocytes secrete pro-inflammatory cytokines that contribute to disease progression [9].

The PLR is determined by dividing the platelet count by the lymphocyte count. Elevated platelet counts indicate increased activation and release of pro-inflammatory and prothrombotic substances from platelets, which promote inflammatory tissue injury and thrombus formation. Lymphocytes, on the other hand, counteract this process through regulatory mechanisms. Compared to platelet or lymphocyte count alone, a higher PLR value has been found to be better at assessing inflammation [10, 11].

The mean platelet volume (MPV) is an index that measures platelet size and activity. Increased MPV value indicates reactive bone marrow thrombocytosis triggered by stress-induced platelet consumption seen in infectious and inflammatory conditions (i.e., sepsis, coronary artery disease, cerebrovascular disease, thrombosis, and chronic inflammatory disorders). Higher MPV values are associated with larger, younger, and functionally hyperactive platelets, and have been linked to a poorer prognosis in septic patients [12,13]. The ratio of MPV to platelet count (MPV/PC) has been introduced as a superior predictor of acute myocardial infarction outcomes compared to MPV alone [14].

In our study, we observed the strongest negative correlation between Hb and PLR, regardless of gender and age. Although the correlation was weak, it remained statistically significant. Santos-Silva et al. has reported a negative correlation between hemoglobin and CRP [7], while platelet counts have been shown to rise during inflammation along with acute phase reactants and pro-inflammatory molecules (i.e., CRP, tumor necrosis factor, interleukin-1 and interleukin-6) [15]. These findings help explain the negative correlation we found between Hb level and PLR.

We also have found a weak negative correlation between Hb level and MLR in males only. Other studies have similarly demonstrated the impact of gender on MLR, with higher MLR values in males compared to females. [16]

When comparing laboratory values between referred and non-referred groups, we found that the referred group had significantly lower Hb levels and significantly higher NLR, MLR, and PLR values. These results are consistent with other studies that found anemia associated with increased hospital admission rates in patients with chronic obstructive pulmonary disease (COPD) [17], as well as higher NLR values in geriatric influenza patients who were not discharged [18]. The elevated MLR and PLR in the referred-to medical ward group also highlight the potential of these inflammatory markers, as previous studies show higher NLR, MLR, and PLR values at admission in deceased coronavirus disease 2019 (COVID-19) patients compared to survivors [19].

Interestingly, we did not find a statistical difference in the MPV/PC ratio between referred and non-referred groups. The prognostic value of MPV in critically ill sepsis patients has been examined in previous studies, with inconsistent findings [5]. Also, in a study by Zampieri et al., the association between elevated MPV levels following intensive care unit (ICU) admission and higher hospital mortality was reported [20]. However, there was no significant difference in baseline MPV levels at the time of admission between survivors and non-survivors [20].

When using ROC analysis to investigate the predictive ability of these ratios regarding referral to the medical ward, we found that the NLR/Hb ratio was the most predictive. In their study, Jheng et al. have shown the predictive ability of NLR for discharge in elderly patients with influenza, using a cutoff of NLR ≤ 6.5 [18]. In our study, NLR/Hb ratio was found to be more predictive than NLR alone for medical ward referral. We also examined the predictive ability of NLR/Hb ratio for the three most common patient groups (digestive, genitourinary, and respiratory), and found that it remained significant in the genitourinary group with a cutoff of 0.289 and in the respiratory group with a cutoff of 0.276. It is crucial to mention that while assessing sex and age as confounders, there was no discernible effect on the final conclusions.

It is also important to acknowledge the limitations of our study, including its monocentric nature, lack of prior research on the exact topic, and relatively small sample size, along with a small duration of the study and the season effect on the resulting prevalences obtained, as our study was done on patients seen in the ED at the end of summer to the end of autumn, and also not taking into account the effect of comorbidities on our results. Future directions could involve investigating the predictive ability of NLR/Hb ratio in larger and more diverse patient populations, as well as exploring its utility in specific disease groups such as pneumonia or pyelonephritis, and also in groups of patients with specific comorbidity such as hypertension or diabetes.

Despite these limitations, the strengths of our study include being the first to demonstrate the importance of NLR/Hb ratio in predicting the referral of patients presenting to the ED and later diagnosed with various diseases, to the medical ward, particularly in the context of genitourinary or respiratory pathologies. Additionally, the retrospective nature of the study allowed us to collect data from a heterogeneous group of 379 patients classified into 14 groups, encompassing a wide range of diseases. Conducting further analysis on patients with homogenous diseases could be an important direction for future research.

## Conclusions

This retrospective study provided evidence of a negative correlation between hemoglobin (Hb) levels and platelet-to-lymphocyte ratio (PLR), highlighting the potential relationship between these two parameters. Moreover, our study identified neutrophil-to-lymphocyte ratio (NLR), monocyte-to-lymphocyte ratio (MLR), and PLR as significant inflammatory markers, consistent with existing literature. Importantly, our study is the first of its kind to demonstrate the predictive value of NLR/Hb ratio for referral to the medical ward following presentation to the emergency department. This finding is particularly relevant for patients in the genitourinary and respiratory groups, underscoring the importance of NLR/Hb ratio as a valuable tool for risk assessment in these patient populations, as a prognostic marker that can reflect the need for admission when the clinical presentation is more severe.
